# Community pharmacists’ perceptions and experiences of medicine shortages in disruptive situations: a qualitative study

**DOI:** 10.1007/s11096-024-01799-7

**Published:** 2024-09-13

**Authors:** Rivana Bachoolall, Fatima Suleman

**Affiliations:** College of Health Sciences, University of KwaZulu-Natal, Durban, KwaZulu-Natal South Africa

**Keywords:** Community pharmacists, Disruptive situations, Medicine shortages, South Africa

## Abstract

**Background:**

Medicine shortages are a challenge in upper, lower and middle-income countries, including South Africa. In recent years, community pharmacists, in Durban, South Africa, have experienced the COVID-19 pandemic, flooding, civil unrest and electricity disruptions. Little is known about the impact of these disruptions on medicine shortages in community pharmacies.

**Aim:**

To explore community pharmacists' perceptions and their experiences with medicine shortages during the COVID-19 pandemic and other disruptive situations.

**Method:**

Convenience and snowball sampling were used to recruit participants. Semi-structured interviews were conducted in person or via an online video conferencing platform, which were audio-recorded and transcribed verbatim. Using the Framework Method, the transcripts were analysed thematically on NVivo 14 software.

**Results:**

Fifteen community pharmacists were interviewed. Five major themes emerged from thematic analysis: general perceptions of medicine shortages, the impact of disruptive situations, the consequences of medicine shortages, mitigation strategies; and further suggestions and resources. Disruptive situations were perceived to exacerbate shortages. Participants perceived a negative financial impact on patients and pharmacies, with out-of-pocket costs affecting the former and loss of income affecting the latter. The mitigation strategies used were contacting stakeholders, medicine substitution and stock management.

**Conclusion:**

Community pharmacists felt that improved communication, collaboration, policies, notification systems and guidelines would mitigate shortages.

**Supplementary Information:**

The online version contains supplementary material available at 10.1007/s11096-024-01799-7.

## Impact statements

- Community pharmacists may experience an increase in medicine shortages during future disruptive situations.
- With the absence of clear guidelines, mitigating medicine shortages in community pharmacies becomes time-consuming and challenging.
- Implementing new legislation, notification systems and guidelines in South Africa and other lower and middle-income countries can help reduce medicine shortages and limit the impact on patients and pharmacies.

## Introduction

Medicine shortages impact patients and healthcare professionals (HCPs) globally [1, 2]. According to the World Health Organization (WHO), medicine shortages occur when the demand for medicines exceeds the supply, which may create a medicine “stock-out” and the patient’s clinical needs are unmet [3]. Consequently, patients face increased out-of-pocket costs. Medicine shortages can lead to poor clinical outcomes, time wastage and negative emotions in patients [4, 5]. Pharmacists in Belgium and Pakistan reported that dealing with medicine shortages was time-consuming and led to a loss of trustworthiness and revenue for the pharmacy [6, 7]. HCPs in a South African community healthcare centre experienced an increased workload and frustration when dealing with medicine shortages [8].

South Africa’s healthcare system comprises the public and the private sectors [9]. Previous South African studies are limited to the public sector and have explored the perspectives of patients and HCPs [8, 10–13]. Researchers in Saudi Arabia, Canada and Australia explored medicine shortages in community pharmacies during normal situations, whereas studies in Pakistan and India were conducted during the COVID-19 pandemic [7, 14, 16, 17]. The COVID-19 pandemic provided a unique circumstance whereby the supply chain was constrained.

The causes of medicine shortages may be due to supply (raw material shortages and problems with production, manufacturing or shipping), demand (increased product demand and rarely, damage to manufacturing sites by natural disasters or inclement weather conditions) or regulatory issues [1, 18]. How pharmacists experience these events and deal with them is unclear.

### Aim

To explore community pharmacists’ perceptions and their experiences with medicine shortages during the COVID-19 pandemic and other disruptive situations.

### Ethics approval

Ethics approval was granted by the University of KwaZulu-Natal’s Biomedical Research Ethics Committee (Reference no: BREC/00005865/2023).

## Method

This was a qualitative, exploratory study. Semi-structured interviews were conducted with community pharmacists in Durban, South Africa, who were identified as information-rich participants. Participants were recruited by a convenience sample, using a snowball technique. Initial participants were invited by e-mail. Subsequent participants were invited using messages, telephone calls or in-person contact. Data collection took place between the 30th of August and the 27th of September 2023.

Semi-structured interviews allowed participants to answer open-ended questions, enabling deeper delving into the research problem [19]. The interview guide (Appendix S1) was adapted from a study by Bogaert et al., who analysed medicine shortages in Belgium and France; and pilot-tested the questions [20]. In-person interviews were conducted alone, in a private area, or via an online video conferencing platform and were audio-recorded. Interviews were conducted until data saturation was reached and no new themes emerged [21]. Field notes were made after each interview. The interviewer (RB) is a female master’s student and a community pharmacist interested in medicine shortages, of which participants were made aware. RB has pharmacy management experience and received training in research ethics and qualitative analysis. Any potential bias in interpreting the data is acknowledged.

**Fig. 1:**
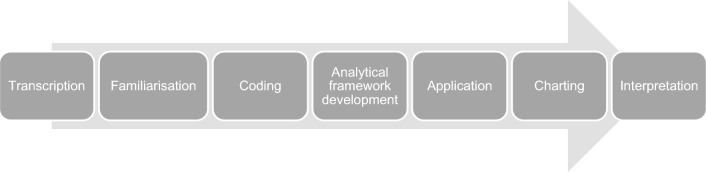
The Framework Analysis method

Thematic analysis was conducted using NVivo 14 software and the Framework Method outlined by Gale et al. [22] (Fig. 1). Interviews were transcribed verbatim, followed by data familiarisation. Codes were assigned deductively. A literature review informed initial codes and themes. A working analytical framework was developed (Appendix S2). Each theme was charted into framework matrices on NVivo 14 and the data was interpreted. The research professor supervising the study (FS) validated the coding.

## Results

Fifteen pharmacists were interviewed, with a further nine people declining participation. Table 1 presents the participant demographics. The median experience as a community pharmacist was 8 years (interquartile range = 6.25). The duration of each interview ranged from 16 to 41 minutes. Five participants were known to the interviewer and the remainder were unknown. None dropped out or requested transcript checking. There was one follow-up interview, due to a recording issue.

**Table 1:** Participant demographics

Characteristics	Options	n
Gender	Male	7
	Female	8
Age	25–34 years old	8
	35–44 years old	0
	45–54 years old	5
	≥ 55 years old	2
Qualification	Bachelor of Pharmacy (Honours) (B.Pharm)	15
Job description	Responsible Pharmacist	7
	Pharmacist	7
	Pharmacy owner	1
Experience in community pharmacy (years)	1–10 years	8
	11–20 years	0
	21–30 years	4
	≥ 31 years	3
Type of community pharmacy	Independent	12
	Corporate	3
Interview method	In-person	12
	Video conference	3

Five themes and eighteen sub-themes were identified.

**Table 2:** Themes and sub-themes and verbatim quotes

Themes	Sub-themes	Verbatim quotes
General perceptions of medicine shortages	Perceived definitions	“When we cannot get a supply of the medicine we require for a patient.” (P1) “Anything that is in demand or need but not readily available to dispense to the patient.” (P9)
	Perceived occurrences of medicine shortages	“I would think approximately at least 10 times in a month, even today. There’s always something that’s out of stock or not available with no alternative.” (P1) “I would rather say undefined or unlimited. Because something can come back after three months, it can back after six months.” (P5) “I would say it varies but from my personal experience… I would say more than a month or indefinitely.” (P7)
	Dynamics of medicine shortages	“I think it’s worsened because every week that I’m ordering, there’s something that’s unavailable, actually, a whole list of things.” (P10) “I’d say with a lot more generics on the market, it hasn’t intensified.” (P3) “One of the companies will go short, and then there’s a knock-on effect, all the other molecules start going out [of stock].” (P14)
	Out-of-stock medicines	“I think pain, pain and inflammation of recent actually.” (P3) “It has been an issue with certain antibiotics.” (P6) “The most we get is on chronic medication. Specifically, anti-hypertensives and I think nowadays some of the anti-diabetic medications.” (P9)
	Perceived causes	“Raw material shortages have become prevalent mainly after COVID.” (P4) “There’s been a lot of [product] discontinuation recently.” (P13)
The impact of disruptive situations	The COVID-19 pandemic	“I actually think COVID started the cascade. Before this, I never really noticed there to be such a major issue with regard to out-of-stocks.” (P13) “Every week there was, depending on what… was trending, people were rushing in to buy that product. Suddenly you go out of stock with it, whether it works or not, we don't know.” (P4)
	Other disruptive situations	“We had unrest, I think it was a year or two ago… there was a major issue with suppliers not being able to meet the demands of the pharmacies… vehicles were stolen, or the wholesalers were ransacked.” (P12) “We did experience the floods and it was quite disastrous. It impacted our supply because the wholesaler couldn’t physically get the stock to us… But the wholesaler managed to re-route via other wholesalers in the country and they did manage to bring in the stock.” (P14)
The consequences of medicine shortages	Cost or financial impact	“If they [medical schemes] said this brand [is covered] and you're using another, they just don't approve it, and then the patient has to pay out-of-pocket.” (P11) “If the patient loses faith in our services… then he will leave our business and go to another pharmacy. So, from a financial point of view, that's like a loss of income as well.” (P7)
	Emotional impact	“The patients get frustrated; they get upset with you.” (P13) “Suddenly an item goes out of stock and they're [the elderly patients] very hesitant, very resistant to change.” (P12)
	Impact on health outcomes	“In many instances, there’s no generic and the patient has to stop taking the drug, compromising their health.” (P4) “There's no continuation of treatment and then you get patients getting sick.” (P9) “The patient doesn't get the care that they are meant to, so… it affects their health, it affects their well-being.” (P14)
	Impact on the roles and responsibilities of a pharmacist	“Patients do not understand why there is a shortage… so it hampers the relationship between patient and pharmacist.” (P7) “The most important consequence is that the patient is not going to receive the care that I took an oath to fulfil.” (P15)
Mitigation strategies	Medicine substitution	“The first is to, like offer generics… Say, for example, the patient's generic is out of stock, and we have to give them the originator.” (P3) “Then [we] look for a therapeutic equivalent.” (P6) “If for example, a slow-release drug is out of stock, and only the plain version is available, we'd have to discuss it as well.” (P10) “We would give the individual ingredients separately, so the patient is not left without any form of treatment.” (P7)
	Contacting stakeholders	“We look at alternative suppliers if we can.” (P15) “We just put it on the WhatsApp group, I'm looking for a particular item.” (P6) “If all else fails, then the doctor has to give an alternative.” (P10) “Once the suppliers tell me it's a manufacturer issue, then I phone the manufacturer.” (P8)
	Stock management	“I think the best way is to keep bulk [stock].” (P2) “We don't want to keep too much money in stock because stock is expensive.” (P6)
Further suggestions and resources	Communication	“They [The manufacturers] don't communicate, that's the biggest problem in this country.” (P1)
	Collaboration	“I think all role-players should be involved.” (P9) “I believe they [the health authorities] should have a more active role… in managing these shortages.” (P7)
	National policy changes	“If something goes wrong in production, even if there's a law, if it goes wrong, it goes wrong. So, I think it'll be a bit hard to bring in laws for that.” (P2) “I think it should be a law… especially when it comes to chronic medication, there needs to be sufficient projection by the [manufacturing] companies to know that they have enough of that medication to service their customers.” (P9)
	Notification systems and guidelines	“I would say the moment there is a medicine shortage… from the [manufacturing] company to the wholesaler, the company needs to issue a letter of shortage, which then needs to reach the pharmacy immediately” (P13) “I believe there should be some form of… database or a system put in place.” (P7) “I do feel… just in the workplace, like implementation of certain or new Standard Operating Procedures (SOPs).” (P8)

The participant number is denoted by (Pn) after each verbatim quote. Words appearing in square brackets denote those that the authors added for context

Each theme describes the verbatim quotes in Table 2.

### General perceptions of medicine shortages

Participants agreed on the definition of medicine shortages and experienced the issue at varying frequencies and durations. There were disparate views regarding the dynamics of medicine shortages in recent years. Participants highlighted a “ripple effect” – when a specific brand of medicine went out of stock, all the other alternative medicines would subsequently go out of stock. Frequently out-of-stock medicines are listed in Table 3.

**Table 3:** Medicines frequently out of stock

Medicine categories	Medicines
Antibiotics	Azithromycin tablets
	Cefpodoxime suspension
	Combination tablets for tuberculosis
	Moxifloxacin tablets
Anti-diabetic medicines	Injectable pre-filled pens such as dulaglutide, liraglutide and semaglutide
	Slow-release metformin tablets
Anti-hypertensive medicines	Amlodipine tablets
	Lercanidipine tablets
Anti-inflammatories	Combination tablets containing naproxen and omeprazole
	Lornoxicam tablets
	Mefenamic acid syrup/suppositories
Anti-migraine medicines	Clonidine tablets
Human growth hormone	Somatropin injection
Topical anaesthetics	Amethocaine cream
	Lidocaine jelly
Smoking cessation aids	Varenicline tablets

Participants highlighted the following causes: raw material shortages, outbreaks, epidemics, pandemics, manufacturing problems, civil unrest, natural disasters, product discontinuation, rationing, quotas and industry consolidation.

### The impact of disruptive situations

Participants agreed that the COVID-19 pandemic created an unexpected demand for medicines. One participant felt that the pandemic created a chain reaction. Changes in prescribing habits and panic buying increased the demand. Participants recalled the civil unrest, which adversely impacted the medicine supply chain. Flooding posed a similar dilemma, and alternative wholesalers had to be utilised.

### The consequences of medicine shortages

Participants highlighted that the main financial impact on patients was out-of-pocket costs. Pharmacies lost income when patients opted to go to another pharmacy. Participants felt that patients experienced frustration, were hesitant to take alternatives and suffered negative health outcomes. Medicine shortages were perceived to impair the pharmacist-patient relationship. One participant highlighted that it impacted the pharmacist’s duty.

### Mitigation strategies

Participants substituted out-of-stock medicines with generic or therapeutic equivalents. Some opted for originator brands, different dosage forms; or split fixed-dose combinations into individual medicines. Participants contacted stakeholders such as wholesalers, other pharmacies and manufacturers. When generics were unavailable, prescribers were called to suggest therapeutic equivalents. Participants mentioned keeping bulk stock as a preventative strategy. However, some felt that this restricted cash flow.

### Further suggestions and resources

Participants agreed that there was insufficient communication regarding medicine shortages. Suggestions included collaboration between stakeholders, including the health authorities. Participants were unaware of laws influencing medicine shortages and some were ambivalent about national policies. The need for out-of-stock letters from manufacturing companies was highlighted, as some felt that word-of-mouth notifications were unreliable. One participant suggested implementing guidelines and another suggested a national database.

## Discussion

### Statement of key findings

Community pharmacists experienced increased medicine shortages during the disruptive situations. Patients were perceived to suffer poor health outcomes and negative emotions. Participants felt that dealing with prolonged medicine shortages experienced during the disruptions was time-consuming and caused patients to lose trust in them, impairing the pharmacist-patient relationship. The perceived financial impact on patients was out-of-pocket costs, whereas pharmacies lost sales. Mitigation strategies were medicine substitution, contacting stakeholders and stock management. These findings were not dissimilar to those experienced during normal working conditions.

### Strengths and weaknesses

This is the first qualitative study to explore medicine shortages in South African community pharmacies. The Consolidated Criteria for Reporting Qualitative Research (COREQ) checklist was used in this study, to ensure credibility. Trustworthiness is displayed through the transparency and consistency of the research steps followed [23, 24]. Recall bias may be a limitation and this study cannot be generalised, as the sample was small and limited to one city. Interviews with pharmacists who suffered property loss due to disruptive situations were omitted from this study.

### Interpretation

Participants had similar explanations for the term “medicine shortages”, unlike the findings of Bogaert et al., who interviewed various stakeholders [20]. Opposing views on the dynamics of medicine shortages in recent years may be attributed to recall bias. The “ripple effect” described is congruent with the “vicious cycle” described by Tan et al. [16]. The perceived reasons for medicine shortages included pandemics, natural disasters and civil unrest. This builds on the previously reported causes under normal conditions [1, 18]. The COVID-19 pandemic was believed to impact the supply chain. The panic buying of medicines aligns with studies in Pakistan and India [7, 17]. This study highlights that civil unrest and flooding were perceived to exacerbate and prolong supply chain issues. However, the impact of electricity disruptions, which may be attributed to adaptations, was not discussed.

Medicine shortages may result in poor financial, emotional and health outcomes for patients. Patients were perceived to pay out-of-pocket costs, in line with prior studies [1, 4, 5]. Pharmacists felt that dealing with medicine shortages was time-consuming. When alternative medicines were unavailable, patients would go to other pharmacies. Comparable outcomes occurred in Belgium and Pakistan, where pharmacists reported an increased workload and a loss of income [6, 7]. The mitigation strategies of contacting other pharmacies via group messaging applications mirror previous findings [8, 12, 15]. In addition to medicine substitution mentioned in previous research, this study highlights dispensing different dosage forms or splitting fixed-dose combinations into individual medicines [15–17]. Bulk stock was perceived to restrict cash flow, similar to the findings of Tan et al. [16].

As reported in previous studies, communication, collaboration and policy changes were suggested [1, 5, 7, 8, 14–16]. Several countries have regulations for mandatory reporting and national medicine shortage websites [25]. The South African public sector has a dedicated website, entitled the “Stop Stockouts Project” and the National Drug Policy of 1996 advocates keeping adequate essential medicines [8]. However, there is still a need for a national database and policies in the private sector. More uniformity between the public and private sectors in South Africa is anticipated, as the country shifts to universal health coverage with the National Health Insurance (NHI) [26].

### Further research

Further national qualitative research on medicine shortages should include a patient perspective; and a quantitative evaluation of which products were most affected.

## Conclusion

Medicine shortages are an ongoing challenge, especially during disruptive situations. More effective communication and collaboration between all stakeholders is recommended. The findings of this study can aid decision-makers in lower and middle-income countries with developing policies, notification systems and guidelines for medicine shortages.

## Supplementary Information

Below is the link to the electronic supplementary material.

Supplementary file1 (DOCX 40 kb)

Supplementary file2 (DOCX 72 kb)
