# Supplementary material for: Community pharmacists’ perceptions and experiences of medicine shortages in disruptive situations: a qualitative study
Source: Int J Clin Pharm. 2024 Sep 13;47(1):210–7. doi: 10.1007/s11096-024-01799-7 (PMC11741986; doi:10.1007/s11096-024-01799-7)
Supplement: Supplementary file 1 — Supplementary file1 (DOCX 40 kb) [file 11096_2024_1799_MOESM1_ESM.docx]

**SUPPLEMENTARY MATERIAL**

**Appendix S1. Semi-structured interview guide**

1. **Introduction**
   1. Purpose and objectives of the study
   2. Informed consent
   3. Voluntary participation
   4. Confidentiality
   5. Permission to audio record
2. **Experience and Demographics**
   1. Gender
   2. Age
   3. Qualification
   4. Job description
   5. Number of years of experience in community pharmacy
   6. Type of community pharmacy – corporate, independent, or franchise
3. **General questions**
   1. What is your understanding of the term “medicine shortages”? Allow the participant to answer and then read out the definition, used for the purpose of this study – The World Health Organization defines a medicine shortage as follows: “when demand exceeds supply at any point in the supply chain and may ultimately create a “stock-out” at the point of appropriate service delivery to the patient if the cause of the shortage cannot be resolved in a timely manner relative to the clinical needs of the patient.”
   2. How often have you been encountering the problem of medicine shortages in your own professional practice? Interviewer to tick off the appropriate box, if applicable.

**Table 1**. Frequency versus Duration of Medicine Shortages^[[1]](#footnote-1)^

| Duration | Frequency | | | | |
| --- | --- | --- | --- | --- | --- |
|  | Every week | Every month | Every 3 months | Every 6 months | Every year |
| Less than 1 week |  |  |  |  |  |
| 1 week to 1 month |  |  |  |  |  |
| More than 1 month |  |  |  |  |  |
| Undefined or unlimited |  |  |  |  |  |

- 1. What are the dynamics of medicine shortages in recent years? Has the problem intensified or decreased?
  2. How do medicine shortages manifest themselves and influence your institution?
  3. What medicines or categories of medicines do you experience the most shortages with?
  4. How much of time do you spend, on average, to resolve medicine shortages?
  5. What are the reasons for medicine shortages in your opinion? Ask what they regard as the principal causes then show Table 2 and ask questions about relevant reasons for the shortages: According to some researchers, there are predictable and unpredictable reasons for medicine shortages. In your opinion what is the influence of each of those factors? Do you think other reasons could be added to this table? Could you rate their importance (1-3)?

**Table 2.** Reasons for Medicine Shortages^[[2]](#footnote-2)^

| Unpredictable Reasons | Predictable Reasons |
| --- | --- |
| - Natural disasters - Manufacturing problems - Raw material shortages - Non-compliance with regulatory standards - Packaging shortages - Unexpected demand - Outbreaks, epidemics, or pandemics - Parallel distribution - Competitive issues - Foreign currency exchange effect - Sovereign issues (financial crisis, debt, default) - Other, e.g., Civil unrest | - Product discontinuation - Industry consolidation (mergers & acquisitions) - Limited manufacturing capacity - Just-in-time inventories - Rationing/quotas - Deliberately induced shortages to manipulate pricing - Market shifts - Launch of a new competitor, new formulation, or expiry of a patent - Other |

- 1. What are the most important consequences of medicine shortages in your opinion? What is the cost/financial impact on patients and the pharmacy?

1. **Legal aspects**
   1. Do you know about any laws and/or regulations that may influence the occurrence of medicine shortages?
   2. Do you think new legal regulations and organizational solutions could be implemented in order to reduce the problem of medicine shortages? Please, address this issue in more detail.
   3. Do you know any examples of such regulations in place in other countries?
   4. Which ones out of the existing legal regulations and organizational solutions could be changed in order to reduce the problem of medicine shortages?
   5. Could the rules of stock management implemented in your organization somehow influence the occurrence of medicine shortages?
2. **Communication**
   1. What do you think about the communication regarding the medicine shortages?
   2. Who should be responsible for the communication regarding the medicine shortages?
   3. When should medicine shortages be announced?
   4. How should medicine shortages be announced?
   5. Are you warned of prospective medicine shortages before they occur?
   6. Do you warn customers of a prospective or an existing medicine shortage? How is this done?
3. **Solutions**
   1. Do the wholesalers or health authorities suggest any solutions to alleviate the problem?
   2. Which specific action do you take when a medicine, required by a patient, is out of stock?
   3. Which specific measures are taken by your institution to reduce or prevent medicine shortages? Are there any other measures, in your view, that could be implemented?
   4. How could other stakeholders in the supply chain contribute to reducing medicine shortages?
   5. In some countries, special committees have been set up to reduce medicine shortages. Do you think such an institution might be useful in South Africa?
4. **Conclusion**
   1. Please may you recommend a colleague who you feel will be interested in participating in this study?
   2. Thank you for your participation. Do you have any questions?

1. *Note.* This table is based on the interview guide for pharmacists from the supplementary material in “A Qualitative Approach to a Better Understanding of the Problems Underlying Drug Shortages, as Viewed from Belgian. French and the European Union’s Perspectives” by Bogaert, P., Bochenek, T., Prokop, A., & Pilc, A., 2015, *PloS one 10*(5)*,* p. 1, Copyright 2015 by Creative Commons Attribution License. [↑](#footnote-ref-1)
2. *Note.* This table is based on the interview guide for pharmacists from the supplementary material in “A Qualitative Approach to a Better Understanding of the Problems Underlying Drug Shortages, as Viewed from Belgian. French and the European Union’s Perspectives” by Bogaert, P., Bochenek, T., Prokop, A., & Pilc, A., 2015, *PloS one 10*(5)*,* p. 2, Copyright 2015 by Creative Commons Attribution License. [↑](#footnote-ref-2)
