# Supplementary material for: Community pharmacists’ perceptions and experiences of medicine shortages in disruptive situations: a qualitative study
Source: Int J Clin Pharm. 2024 Sep 13;47(1):210–7. doi: 10.1007/s11096-024-01799-7 (PMC11741986; doi:10.1007/s11096-024-01799-7)
Supplement: Supplementary file 2 — Supplementary file2 (DOCX 72 kb) [file 11096_2024_1799_MOESM2_ESM.docx]

**Appendix S2. Themes and Codes**

Deductive coding was employed in this study. The tables below show the initial and final themes and sub-themes, followed by notes on the changes.

**Table 1.** Initial Themes and Sub-themes

| Themes | Sub-themes |
| --- | --- |
| 1. Perceptions and dynamics of medicine shortages | 1. Perceived definitions 2. Frequency and duration of medicine shortages 3. Dynamics of medicine shortages 4. Medicines and medicine categories 5. Causes |
| 1. Impact of disruptive situations | 1. The COVID-19 pandemic 2. Other disruptive situations |
| 1. Cost or financial impact | 1. Out-of-pocket costs 2. Travel costs 3. Medicine costs 4. Staff costs |
| 1. Mitigation strategies | 1. Contacting suppliers 2. Contacting other pharmacies 3. Contacting the prescriber 4. Generic substitution 5. Originator brand substitution 6. Therapeutic substitution |
| 1. Further suggestions and resources | 1. Collaboration and communication 2. Notification, reporting and tracking systems 3. National policy changes and guidelines 4. Other suggestions |

**Table 2.** Final Themes and Sub-themes

| Themes | Sub-themes |
| --- | --- |
| 1. General perceptions of medicine shortages | 1. Perceived definitions 2. Perceived occurrences of medicine shortages 3. Dynamics of medicine shortages 4. Out-of-stock medicines 5. Perceived causes |
| 1. Impact of disruptive situations | 1. The COVID-19 pandemic 2. Other disruptive situations |
| 1. Consequences of medicine shortages | 1. Cost or financial impact 2. Emotional impact 3. Impact on health outcomes 4. Impact on the roles and responsibilities of a pharmacist |
| 1. Mitigation strategies | 1. Medicine substitution 2. Contacting stakeholders 3. Stock management |
| 1. Further suggestions and resources | 1. Communication 2. Collaboration 3. National policy changes 4. Notification systems and guidelines |

**Notes on the Changes to the Themes and Sub-themes:**

- Initially, there were 5 themes and 21 sub-themes. After the qualitative data was coded on NVivo 14 Software and the Framework Method was applied, there were 5 final themes and 18 final sub-themes.
- Theme 1 - “Perceptions and dynamics of medicine shortages” was renamed “General perceptions of medicine shortages.”
- The sub-theme - “Frequency and duration of medicine shortages” was renamed “Perceived occurrences of medicine shortages.”
- The sub-theme – “Medicines and medicine categories” was renamed “Out-of-stock medicines.”
- Theme 3 – “Cost or financial impact” was replaced with “Consequences of medicine shortages,” to include the original theme and non-financial consequences.
- The initial sub-themes – “Out-of-pocket costs,” “Travel costs,” “Medicine costs” and “Staff costs” were replaced with “Cost or financial impact,” “Emotional impact,” “Impact on health outcomes” and “Impact on the roles and responsibilities of a pharmacist.”
- The initial sub-themes – “Contacting suppliers,” “Contacting other pharmacies” and “Contacting the prescriber” were consolidated into the sub-theme “Contacting stakeholders.”
- The initial sub-themes – “Generic substitution, “Originator brand substitution” and “Therapeutic substitution” were consolidated into the sub-theme “Medicine substitution.”
- A third sub-theme, “Stock management,” was added to the fourth theme, “Mitigation strategies”
- The initial sub-theme – “Collaboration and communication” was split into two separate sub-themes, “Communication” and “Collaboration”.
- The initial sub-theme – “Notification, reporting and tracking systems” was renamed “Notification systems and guidelines.”
- The initial sub-theme – “National policy changes and guidelines” was renamed “National policy changes.”
- The initial sub-theme – “Other suggestions” was removed, as the coding fell within the final “Collaboration” sub-theme.

**Table 3.** Initial Codebook

| Theme | Sub-theme | Code |
| --- | --- | --- |
| 1. Perceptions and Dynamics of Medicine Shortages^1,2^ | Causes^1-7^ | Other Causes^1^  Predictable Causes^1^  Unpredictable Causes^1^ |
|  | Dynamics of Medicine shortages^1^ | Decreased^1^  Intensified^1,5,7^ |
|  | Frequency and Duration of Medicine Shortages^1^ | Duration^1,5,8^  Frequency^1,3,5,6^ |
|  | Medicines and Medicine Categories^1,2,4,9^ | Categories^1,2,4,6,9^  Medicines^1,2,4,8,10^ |
|  | Perceived Definitions^1,4^ | Definition^1,4^ |
| 1. Impact of Disruptive Situations^2,3,9^ | The COVID-19 Pandemic^2,9^ | COVID-19^2,9^ |
|  | Other Disruptive Situations^3^ | Civil Unrest^10,11^  Natural Disasters^1,3,12^  Load Shedding^13^ |
| 1. Cost or financial impact^4,14^ | Patient^4,5,7,10^ | Medicine Costs^3,4,10^  Out-of-pocket Costs^3,4,7,10,14^  Travel Costs^9,10,14^ |
|  | Pharmacy^2,3,7,9,15^ | Loss of Sales^2,9,15^  Staff Costs^3,5^ |
| 1. Mitigation Strategies^4,7,16^ | Contacting stakeholders | Contacting Other Pharmacies^4,6,9,16^  Contacting Suppliers^4,9,16,17^  Contacting the Prescriber^4,6,9,17^ |
|  | Medicine Substitution^2-4,6,8^ | Generic Substitution^2,15,16^  Originator Brand Substitution^16^  Therapeutic Substitution^7,16^ |
| 1. Further Suggestions and Resources^2-5,7,10,15,17^ | Collaboration and Communication^2-5,7,10,15^ | Collaboration^2,3,5,7,15^  Communication^2-4,7,10,15^ |
|  | Notification, Reporting and tracking systems^3-5,7,10,17^ | Notification Systems^3,4,7^  Reporting Systems^4,5,10,17^  Tracking Systems^3,4,17^ |
|  | National Policy Changes and Guidelines^3,4,15,17^ | Guidelines^4,17^  Policy Changes^3,4,15,17^ |
|  | Other suggestions | Other |

The following references, for the above table, were consulted to provide the initial themes, sub-themes and codes.

**References**

1. Bogaert P, Bochenek T, Prokop A, et al. A qualitative approach to a better understanding of the problems underlying drug shortages, as viewed from Belgian, French and the European Union’s perspectives. PloS One. 2015;10(5):e0125691.
2. Omer S, Ali S, Shukar S, et al. A qualitative study exploring the management of medicine shortages in the community pharmacy of Pakistan. Int J Environ Res Public Health. 2021;18(20):10665.
3. Fox ER, Sweet BV, Jensen V. Drug shortages: a complex health care crisis. Mayo Clin Proc. 2014;89(3):361-73.
4. Shukar S, Zahoor F, Hayat K, et al. Drug shortage: causes, impact, and mitigation strategies. Front Pharmacol. 2021;12:693426.
5. Rinaldi F, de Denus S, Nguyen A, et al. Drug shortages: patients and health care providers are all drawing the short straw. Can J Cardiol. 2017;33(2):283-6.
6. Heiskanen K, Ahonen R, Karttunen P, et al. Medicine shortages--a study of community pharmacies in Finland. Health Policy. 2015;119(2):232-8.
7. Tan YX, Moles RJ, Chaar BB. Medicine shortages in Australia: causes, impact and management strategies in the community setting. Int J Clin Pharm. 2016;38(5):1133-41.
8. Hwang B, Shroufi A, Gils T, et al. Stock-outs of antiretroviral and tuberculosis medicines in South Africa: A national cross-sectional survey. PloS One. 2019;14(3):e0212405.
9. Ramakrishnan M, Poojari PG, Rashid M, et al. Impact of COVID-19 pandemic on medicine supply chain for patients with chronic diseases: Experiences of the community pharmacists. Clin Epidemiol Glob Health. 2023;20:101243.
10. Ndzamela, S. Patients and healthcare professionals’ experiences of medicine stock-outs and shortages at a community healthcare centre in the Eastern Cape. S Afr Pharm J. 2020;87(5):18-22.
11. Robertson E. Venezuelan unrest increases pressure on health services. Lancet. 2014;383(9921):942.
12. Melin K, Rodríguez-Díaz CE. Community pharmacy response in the aftermath of natural disasters: time-sensitive opportunity for research and evaluation. J Prim Care Community Health. 2018;9:2150132718813494.
13. Shah SZA., Shaikh MK, Nisar N, et al. The health consequences of power outages - electricity load-shedding problem in country. Pak J of Med & Health Sci, 2023;17(2):1
14. Phuong JM, Penm J, Chaar B, et al. The impacts of medication shortages on patient outcomes: a scoping review. PloS One. 2019;14(5):e0215837.
15. Atif M, Sehar A, Malik I, et al. What impact does medicines shortages have on patients? A qualitative study exploring patients’ experience and views of healthcare professionals. BMC Health Serv Res. 2021;21(1):827.
16. Panic G, Yao X, Gregory P, et al. How do community pharmacies in Ontario manage drug shortage problems? Results of an exploratory qualitative study. Can Pharm J (Ott). 2020;153(6):371-7.
17. Alsheikh MY, Alzahrani MA, Alsharif NA, et al. Community pharmacy staff knowledge, opinion and practice toward drug shortages in Saudi Arabia. Saudi Pharm J. 2021;29(12):1383-91.

**Final Codebooks** – Generated on NVivo 14

**Table 4.1** Theme 1 - General Perceptions of Medicine Shortages

| Name | Description |
| --- | --- |
| **Dynamics of Medicine Shortages** | |
| Manifestation | How medicine shortages arise. |
| ***Recent Years*** | |
| Decreased | Medicine shortages have decreased in recent years. |
| Evolution | How medicine shortages have evolved over the years. |
| Intensified | Medicine shortages have increased in recent years. |
| Ripple Effect | When one medicine goes out of stock and causes an expected demand on other generics, which subsequently go out of stock. |
| **Perceived Occurrences of Medicine Shortages** | |
| Duration | How often medicine shortages occur. |
| Frequency | The period over which medicine shortages occur. |
| **Out-of-stock Medicines** | |
| Categories | Classes or groups of medicines, e.g. anti-diabetic medicines, anti-hypertensive medicines and analgesics. |
| Medicines | Specific medicine names, whether brand or active ingredients. |
| **Perceived Causes** | |
| ***Other*** | |
| Economy | The economic state of the country. |
| Global Warming | Increasing temperatures worldwide. |
| Lack of Staff | An insufficient number of skilled workers. |
| Off-label Use | The use of a medicine for an indication not listed on the package insert. |
| Prescribing Habits | The tendency of doctors to prescribe certain medicines. |
| Public-Private Partnerships | Collaboration between the public and private sectors of healthcare. |
| Suez Canal Blockage | The blockage of the Suez Canal by a container ship in 2021. |
| War | State of conflict between or within countries. |
| ***Predictable Causes*** | |
| Deliberately Induced Shortages to Manipulate Pricing | When shortages are intentional to make the price higher. |
| Industry Consolidation | Mergers and Acquisitions, including the repacking or rebranding |
| Just-in-time Inventories | Ordering just enough stock for a limited number of day’s supply |
| Launch of a New Competitor, New Formulation or Expiry of a Patent | When pharmaceutical companies introduce a new medicine or formulation onto the market, to compete with existing medicines, or when the period a patent on a medicine elapses. |
| Limited Manufacturing Capacity | The reduced ability to manufacture large volumes of medicines. |
| Market Shifts | Changes in the sales or demand of medicines. |
| Product Discontinuation | When a medicine is withdrawn from the market and is no longer available. |
| Rationing or Quotas | Allocating limited or specific amounts of medicines to avoid running out. |
| ***Unpredictable Causes*** | |
| Civil Unrest | The stealing of goods during riots. |
| Competitive Issues | When companies in the supply chain compete with one another. |
| Foreign Currency Exchange Effect | The effect of the exchange rate on medicine shortages. |
| Manufacturing Problems | When manufacturers experience problems in the production line. |
| Delayed Release | The timing in which the excipients or the batch of medicines is slower than usual |
| Lack of Profitability | Not making enough of a profit on a product. |
| Limited Batch Release | When fewer batches of a medicine are manufactured. |
| Moving Location | When a manufacturer changes the geographic area of the production site. |
| Poor Forecasting | Not planning or predicting the demand for a medicine. |
| Natural Disasters | A disruptive event such as flooding, earthquakes, etc. |
| Non-Compliance with Regulatory Standards | Not meeting the standards required by the health authority. |
| Batch Recall | When a batch is defective and has to be sent back to the supplier. |
| Outbreaks, Epidemics or Pandemics | An outbreak of an infectious disease locally, in a specific geographic area or worldwide. |
| Packaging Shortages | Insufficient availability of the material used to pack medicines. |
| Parallel Distribution | The authorised supply of medicines from one country to another. |
| Raw Material Shortages | Shortage of raw materials or the API (active pharmaceutical ingredient) |
| Sovereign Issues | Government or national debt issues. |
| Unexpected Demand | When medicines are required at a higher rate than the supply. |
| Perceived Definitions | |
| What participants perceive as a definition for “medicine shortage” | |

**Table 4.2** Theme 2 - Impact of Disruptive Situations

| Name | Description |
| --- | --- |
| **Other Disruptive Situations** | |
| Flooding | The overflow of water, as a result of inclement weather conditions. |
| Load Shedding | The rationing of electricity due to limited resources. |
| Looting | The stealing of goods during riots. |
| **The COVID-19 Pandemic** | |
| The Coronavirus pandemic that originated in 2019. | |

**Table 4.3** Theme 3 - Consequences of Medicine Shortages

| Name | Description |
| --- | --- |
| **Cost or Financial Impact** | |
| Medicine Costs | The expense of paying for medicines. |
| ***Patients*** | |
| Blood Tests | Testing a sample of blood for diagnostic purposes. |
| Courier Costs | The cost of using courier services for medicines. |
| Doctor Consults | The cost of going to a doctor for a consultation. |
| Out-of-pocket Costs | Co-payments incurred by the patient on a medical aid. |
| Telephone Costs | The cost of making telephone calls to source medicines. |
| Travel Costs | Fuel or transportation costs incurred by the patient. |
| ***Pharmacy*** | |
| Delivery Costs | The cost of delivery of medicines to a patient. |
| Loss of Income | A reduction in the revenue of the pharmacy. |
| Time Investment | The duration of time spent on dealing with medicine shortages. |
| **Emotional Impact** | |
| Hesitancy and Confusion | When a patient displays scepticism and uncertainty. |
| Patient Dissatisfaction | When a patient is unhappy with the services provided by the pharmacy. |
| Stress and Frustration | A negative emotional response to medicine shortages. |
| **Impact on Health Outcomes** | |
| Non-compliance due to Refusal to use Alternates | Non-adherence to prescribed medicine regimens due to lack of substitutable alternatives |
| Side Effects | Experiencing any untoward or adverse effects from taking a medicine. |
| Unavailability of Alternate Medicines | The lack of alternate medicine to substitute an out-of-stock medicine. |
| **Impact on the Roles and Responsibilities of a Pharmacist** | |
| Medical Aid Issues | Any challenges faced by pharmacists with medical aids. |
| Pharmacist-Patient Relationship | The relationship between the pharmacist and patient. |

**Table 4.4** Theme 4 - Mitigation Strategies

| Name | Description |
| --- | --- |
| **Contacting Stakeholders** | |
| Contacting Other Pharmacies | Communication with other pharmacies. |
| Phoning | Making telephone calls. |
| WhatsApp Groups | Making use of the group function on WhatsApp |
| Contacting the Manufacturer | Contacting the manufacturer of the medicines. |
| Contacting the Prescriber | Contacting the doctor who prescribed the out-of-stock medicines. |
| Contacting Wholesalers | Contacting various wholesalers or suppliers of the medicines. |
| **Medicine Substitution** | |
| Alternative Pack Sizes or Dosage Forms | Using a different pack size or dosage form, in place of the one prescribed. |
| Generic Substitution | Substituting with a medicine that has the same active ingredient and strength. |
| Originator Brand Substitution | Substituting with the brand that was first released onto the market and is often more expensive, compared to generic medicines. |
| Substitution of a Fixed-Dose Combination with Individual Medicines | Separately dispensing the ingredients of a medicine with more than one active ingredient. |
| Therapeutic Substitution | Substituting with an alternative medicine, upon consulting the prescriber. |
| **Stock Management** | |
| ***Procurement Strategies*** | |
| Access to Multiple Wholesalers or Branches | Having accounts with multiple wholesalers or branches of wholesalers. |
| Buffer or Bulk Stock | Ordering in and keeping back-up or bulk stock. |
| Space Constraints | Not having enough space to store buffer or bulk stock. |
| Forecasting | Estimating the anticipated usage of medicines. |
| Ordering Frequency | How often stock orders are placed. |
| Ordering System | The software used to place the medicine order. |
| Responsible Person | The person responsible for placing stock orders. |
| Stock Days | The number of days’ stock on hand kept in a pharmacy. |
| Stock Delivery Frequency | How often medicines are delivered to pharmacies by wholesalers. |
| ***Rationing of Medicines*** | |
| Dispensing medicines according to a need’s basis or severity of disease. | |

**Table 4.5** Theme 5 - Further Suggestions and Resources

| Name | Description |
| --- | --- |
| **Collaboration** | |
| ***Between Stakeholders*** | |
| DOH | Department of Health |
| Manufacturers | The companies responsible for the production of medicines. |
| Medical Representatives | Representatives employed by pharmaceutical companies to market the medicines to healthcare professionals. |
| Other Pharmacies | Neighbouring or other pharmacies within a chain. |
| Owners | The person or company to whom the pharmacy belongs. |
| Pharmacy Council | The South African Pharmacy Council. |
| Prescribers | Doctors, dentists or any authorized persons who can prescribe medicines. |
| SAPHRA | South African Health Products Regulatory Authority |
| Solutions Suggested | Current solutions suggested by health authorities or wholesalers. |
| Wholesalers | The suppliers responsible for the delivery of medicines to pharmacies. |
| *Committees* | |
| Ambivalent | Unsure whether the implementation of a committee will work. |
| Negative | Certain that the implementation of a committee will not work. |
| Positive | Certain that the implementation of a committee will work. |
| Communication | |
| Current Situation | The current situation regarding communication of medicine shortages. |
| *Recommended Communication* | |
| Method of Communication | How medicine shortages are made known to pharmacists. |
| Responsible Party | The person/s responsible for the communication of medicine shortages. |
| Time of Announcement | When medicine shortages should be announced. |
| *Warnings* | |
| To Patients | How pharmacists communicate with patients about medicine shortages. |
| Call | Telephonic communication. |
| Face-to-face | In-person, verbal communication. |
| Message | Communication via WhatsApp or short message service (SMS) |
| To Pharmacies | How pharmacists receive communication regarding medicine shortages. |
| National Policy Changes | |
| Existing Knowledge | Awareness of local laws about medicine shortages. |
| Other Countries | Awareness of international laws about medicine shortages. |
| *Suggested* | |
| Communication | Laws involving the communication of medicine shortages. |
| Distribution | Laws involving the distribution of medicines. |
| Fines | Imposing charges on those who do not comply with legislation. |
| Formulary Changes | Changes to the listing of medicines covered by medical aids. |
| Manufacturing | Laws involving the manufacturing of medicines. |
| Opening a Pharmacy or Wholesaler | Laws regarding the registration and opening of a pharmacy or a wholesaler. |
| Ownership | The person/s who is allowed to own a pharmacy. |
| Patent Laws | The laws regarding the patent of a medicine. |
| Pricing | The cost of medicines. |
| Registration Time Limit | The amount of time it takes to register a new medicine. |
| Scope of Practice | The tasks that can be fulfilled as a pharmacist as per legislation. |
| *Views on New Legislation* | |
| Negative | Participants display a negative view towards implementing new laws. |
| Positive | Participants display a positive view towards implementing new laws. |
| Notification Systems and Guidelines | |
| National Database or Website | The use of an internet-based service to communicate medicine shortages nationally. |
| Out of Stock Letters, Circulars or Lists | Official letters from the manufacturers stating that a medicine is out of stock. |
| SOPs | Standard Operating Procedures |
